# Spatio-Temporal Dynamics of Foraging Networks in the Grass-Cutting Ant *Atta bisphaerica* Forel, 1908 (Formicidae, Attini)

**DOI:** 10.1371/journal.pone.0146613

**Published:** 2016-01-11

**Authors:** Juliane F. S. Lopes, Mariana S. Brugger, Regys B. Menezes, Roberto S. Camargo, Luiz Carlos Forti, Vincent Fourcassié

**Affiliations:** 1 Programa de Pós Graduação em Ciências Biológicas, Comportamento e Biologia Animal, Universidade Federal Juiz de Fora, Campus Universitário de Martelos, Juiz de Fora, Minas Gerais, Brazil; 2 Programa de Pós Graduação em Ecologia, Universidade Federal Juiz de Fora, Campus Universitário de Martelos, Juiz de Fora, Minas Gerais, Brazil; 3 Departamento de Produção Vegetal, Faculdade de Ciências Agronômica, Universidade Estadual Paulista Júlio de Mesquita Filho, Botucatu, São Paulo, Brazil; 4 Centre de Recherches sur la Cognition Animale, Université Paul Sabatier, Université Fédérale de Toulouse Midi-Pyrénées, Toulouse, cedex 9, France; 5 CNRS—Centre de Recherches sur la Cognition Animale, UMR 5169, 118 route de Narbonne, Toulouse, cedex 9, France; Arizona State University, UNITED STATES

## Abstract

Foraging networks are a key element for ant colonies because they facilitate the flow of resources from the environment to the nest and they allow the sharing of information among individuals. Here we report the results of an 8-month survey, extending from November 2009 to June 2010, of the foraging networks of four mature colonies of *Atta bisphaerica*, a species of grass-cutting ant which is considered as a pest in Brazil. We found that the distribution of foraging effort was strongly influenced by the landscape features around the nests, in particular by the permanently wet parts of the pasture in which the nests were located. The foraging networks consisted of underground tunnels which opened on average at 21.5m from the nests and of above-ground physical trails that reached on average 4.70m in length. The use of the foraging networks was highly dynamic, with few sections of the networks used for long periods of time. Three different phases, which could be linked to the seasonal change in the local rainfall regime, could be identified in the construction and use of the foraging networks. The first phase corresponded to the beginning of the rainy season and was characterized by a low foraging activity, as well as a low excavation and physical trail construction effort. The second phase, which began in February and extended up to the end of the humid season at the end of March, was characterized by an intense excavation and trail construction effort, resulting in an expansion of the foraging networks. Finally, in the third phase, which corresponded to the beginning of the dry season, the excavation and trail construction effort leveled off or decreased while foraging activity kept increasing. Our hypothesis is that ants could benefit from the underground tunnels and physical trails built during the humid season to maintain their foraging activity at a high level.

## Introduction

A lot of animals travel inside their home range by using well-defined routes that are cleared of vegetation and obstacles [1]. When these routes branch or interconnect they end up forming a network that facilitates the flow of resources through the environment and the sharing of information among individuals [2]. Foraging networks, i.e. the networks of routes used to transport food, are particularly well documented in ants [3, 4]. In some ant species, particularly in seed-harvesting and leaf-cutting ants [5, 6], these networks are formed of physical trails, called trunk-trails, that are conspicuous enough to be followed on the ground even in the absence of traffic. These trails are not the consequence of the passage of a multitude of ants that would trample the vegetation and passively push the obstacles off the trails but they are the result of an active process. Indeed, as engineers of ecosystems [7], ants build and maintain their routes by cutting the growing vegetation and clearing the debris of various sorts falling on the trails [8]. These routes generally last a few days or up to a few weeks [9–11] but in some ant species, for which the resources exploited are highly persistent and renewed from one year to the other, they can last for years [12–14] and therefore be considered as a "physical memory" of the resource location [15, 16]. The networks typically have a dendritic shape, with one or several trails departing from the nest and branching successively in different directions until reaching the foraging resources.

The main function of trunk-trails in ants is to guide the foragers to resource patches and to allow their fast return to the nest [5, 17]. In fact, because they act as tactile guidelines for the ants, trunk-trails decrease the probability for the ants to lose their way. Most of the scout ants in search of new forage depart directly from the trunk-trails, not from the nest [18]. This allows them to search a more extensive area. Moreover, since scout ants come back to the point where they have left the trail, instead of going back directly to the nest, they are able to mobilize a huge quantity of workers rapidly through chemical recruitment. Physical trails also channel ant traffic and thus increase local interactions and the exchange of information between individuals [19, 20]. In addition, they offer a smooth terrain compared to the surrounding environment and thus allow ants to increase their speed significantly [21] and to transport their loads more easily. Finally, physical trails delimit the foraging areas of neighboring colonies and thus reduce the likelihood of agonistic encounters between individuals of different colonies [22].

Leaf-cutting ants of the genus *Atta* are known to build extensive networks of foraging trails [23] and to cause enormous damage to cultivated plants [24]. Their foraging system is based on the construction and maintenance of physical trails that are 3–5cm in width and that can reach a maximum length of 50m. Once built, these trails can persist for up to eight months without regrowth of vegetation, even when foraging activity has ceased totally [21]. In mature colonies of some *Atta* species such as *A*. *sexdens* [25], *A*. *laevigata* [26] and *A*. *bisphaerica* [27] the network of foraging trails is complemented by underground tunnels that can be up to 70m in length and 5cm in diameter and that connect the nest to distant foraging holes from which one or several trails depart to the resource patches. The combination of underground tunnels and long and persistent physical trails connected to more ephemeral short trails allows a very efficient collection of the vegetation around the nest [25, 28].

Because of the economic importance of leaf-cutting ants, there is a huge literature on their foraging ecology, especially on the species of the genus *Atta* (review in [23]). However, compared to ants that cut dicotyledons [11, 16, 25, 29], relatively few studies have investigated over a substantial period of time the spatial and temporal dynamics of foraging networks in the *Atta* species that cut monocotyledons, i.e. grass-cutting ants. The distribution of monocotyledons is generally much less aggregated than that of dicotyledons, the more so in agropastoral landscapes which, with their low plant diversity, are much more homogeneous than natural landscapes. Therefore, one could hypothesize that grass-cutting ant species should rely less on long lasting trunk-trails and build more ephemeral structures to collect resources than leaf-cutting ant species.

In this paper we describe the spatio-temporal dynamics of the construction and use of physical foraging structures over several consecutive months in the grass-cutting ant *A*. *bisphaerica* Forel, 1908. This species occurs exclusively in Brazil [30, 31] where it has a considerable economic importance because of the damages it causes to some crop plants cultivated on a wide scale, e.g. sugar cane and corn. The nests of *A*. *bisphaerica* are also common in pastures where these ants compete with cattle for grass consumption and also favor the emergence of invasive species [27, 28]. In our study we surveyed the spatial distribution of foraging holes and physical foraging trails of four mature nests of *A*. *bisphaerica* located in the same pasture over an 8-month period, covering the whole wet season and part of the dry season. This allowed us to follow the growth of the foraging networks throughout two seasons characterized by contrasting meteorological conditions, to monitor the use of the foraging structures (underground tunnels, physical trails) they build, and to investigate the way they distribute their foraging effort around their nest. The results show that the foraging networks of *Atta bisphaerica* are highly dynamic and are partly shaped by the landscape features around the nests which influence the distribution of the resources being collected.

## Material and Methods

### Studied colonies and their location

**Fig 1:**
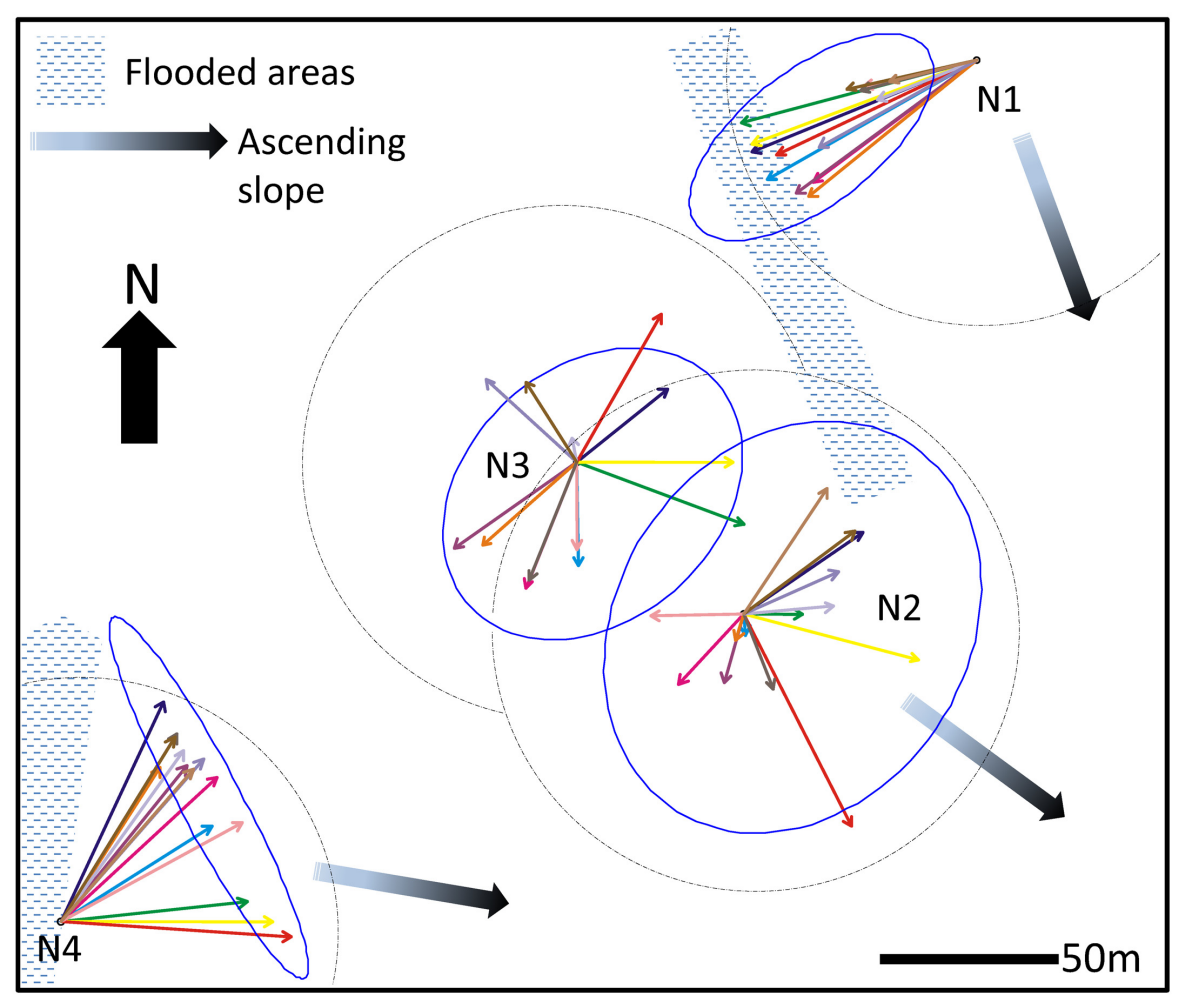
Map of the studied area showing the respective location of the nests and the distribution of foraging effort around the nests throughout the 8-month survey.

The studied nests were located in a privately owned pasture (Sertão Farm, municipality of Coronel Pacheco, south of the state of Minas Gerais, Brazil—21° 39'S, 43° 21'W, altitude: 800m). The study was conducted on private land whose owners kindly allowed its use. The pasture had a gentle slope ascending in the eastern direction, varying in intensity between 12° and 17° (Fig 1 & S1 Fig). It was predominantly covered with *Paspalum notatum* with some patches of *Brachiaria* sp. here and there.

We selected four mature colonies of *Atta bisphaerica* (N1, N2, N3, N4) and placed a stake at the center of the mound of loose soil above each nest at the beginning of the survey. The area covered by loose soil above the nests varied between 50 and 72m^2^ (Table 1). Nest 3 and nest 4 were located down the slope of the pasture and nest 1 and nest 2 were located at mid-slope, about 10 meters in altitude above the level of nest 3 and 4. A water tank was located about 100 meters south of the position of nest 1 and water trickled down the slope in the N-NW direction. The area west of nest 4 was permanently flooded (Fig 1).

**Table 1:** Characteristics of the Four Nests Studied.

Nest	Area of loose soil above the nests (m^2^)^a^		Area growth (%)
	Nov 2009^b^	June 2010^c^	
N1	72.19	130.42	80.66
N2	49.86	85.26	71.00
N3	57.70	64.72	12.17
N4	52.87	83.99	58.86

^*a*^ The area was approximated as the shape of an ellipse.

^*b*^ Beginning of the survey.

^***c***^ **End of the survey**

The positions of the nests in the pasture are shown to scale. For each nest, each arrow corresponds to the mean vector of the direction of the end points of the active foraging trails observed at each visit (see S2, S3, S4 and S5 Figs for the dates of each visit). The dashed line circle around each nest position corresponds to vector unity. The Hotelling 95% confidence ellipse of the directions of the end points of active foraging trails is plotted as a solid line for each nest.

### Mapping and monitoring of the foraging networks

To map the foraging networks, we inspected the area surrounding the nests at intervals of approximately 15 days (mean ± SD: 16 ± 8, range: 3–37) during an 8-month period extending from November 24^th^ 2009 to June 22^nd^ 2010. This period covers most of the wet season (November–March) and part of the dry season (April–October) in the studied area. Because the four studied nests could not be surveyed during the same day, the date of visits varies from one nest to the other (see S2, S3, S4 and S5 Figs for the dates of each visit). The number of visits for each nest was 14, for nests N1, N2, N4 and 13 for nest N3. Each visit began in the morning between 06:00 and 11:00 and, if necessary, was extended in the afternoon, between 15:00 and 18:00. This covers most of the diurnal activity of *A*. *bisphaerica* in the wet season and the end and beginning of the nocturnal activity during the dry season [32].

At the beginning of each visit we checked for new foraging holes with foraging activity and marked them with a labeled stake. A hole was considered as active whenever we could see ants exiting or entering it with pieces of grass, whether or not a physical trail was active. To identify the nest of origin of new foraging holes we used the method developed by Fowler et al. [33] which consists in placing tiny colored objects daubed with powder made from sugar cane leaves around a foraging hole and checking for their presence on the mound of loose soil above the surrounding nests during the next days. We followed all physical trails departing from new holes and marked the end of each trail (including branched trails) with a labeled stake. We then proceeded by inspecting all foraging holes labelled in previous visits, marked the end of all new physical trails (including those branching from or prolonging old trails), if any, departing from these holes and measured their length. If there were physical trails departing from a hole that had been labelled during previous visits, we also noted whether they were active or not. After each visit to the field the position of the new stakes was measured by using a triangulation procedure based on reference points surrounding the nests. These positions, as well as the approximate outlines of each new trail, were reported on a map drawn to scale with the software CorelDraw X4. Note that this sampling procedure excludes the foraging holes opened before the survey that were not active during the first day of sampling and that completely ceased their activity afterwards.

### Data analysis

The development of the foraging network over the survey period was investigated by assessing both the effort put in building underground tunnels and that in building new physical trails. The assessment of excavation effort was based on the number of new active foraging holes that appeared at each visit, whereas the assessment of trail construction was based on the number of new physical trails (departing from foraging holes or from existing trails through branching or prolongation) that appeared at each visit, as well as on the total length of these trails. Note that most foraging holes probably open at the end of short tunnels built through the branching of existing tunnels, and not at the end of newly built tunnels departing directly from the nest. To evaluate the overall foraging activity, we considered the number of active foraging holes and active physical trails at each visit. Finally, the dynamics of foraging network use were investigated by measuring the fraction of foraging holes and of the total length of physical foraging trails reused at each visit. The change over time of each variable measured was assessed by fitting a cubic regression spline to the data with a Generalized Additive Mixed Model (GAMM) [34]. Nest identity was introduced in the model as a random variable. To correct for overdispersion, we used a quasipoisson model for numbers (foraging holes and active trails) and a quasibinomial model for proportions (proportion of old holes or of existing trails reused). The function *gamm* of the R *mgcv* package [35] was used to fit the model.

To assess the amount of area covered by foraging ants throughout the wet and dry season and over the 8-month observation period, we used the maps of the foraging areas created with CorelDraw X4. A grid formed by 0.5m x 0.5m cells was superimposed on each network map and we counted the number of cells occupied by the network. We then multiplied the number of cells by 0.25 to obtain the area covered by the network in m^2^.

The spatial distribution of foraging effort around the nests throughout the observation period was assessed by measuring for each visit the direction of the end point of each active trail in relation to the nest and by using circular statistics to calculate a mean vector of the distribution of these directions [36]. This distribution was then tested against uniformity by using the Rayleigh test of uniformity [36]. To measure any bias in the distribution of the foraging effort around the nests over the whole survey period we used second-order statistics and calculated a Hotelling confidence ellipse [36] with a 95% confidence interval. The ellipse gives the area in which the position of the center of mass of all end points of the foraging trails used throughout the observation period is located with 95% confidence. If the nest is not included in the ellipse this means that the activity is biased towards a certain angular sector [37]. We also tested whether there was any consistent change in the direction of foraging activity around each nest over the survey period [38–40] by plotting over time the angle of the mean vector of the direction of the end point of each active trail at each visit.

All analyses were carried out and graphs generated with the software R 2.13.1.

## Results

### Foraging network overall characteristics

Throughout the 8-month survey, ants opened 459 foraging holes and built 833 physical trails, yielding a total of 2,629 meters of trails for the four nests studied, covering an area of 1,346 m^2^(Table 2). When a foraging hole was active there was only one active foraging trail departing from it in 80% of the cases. Most foraging trails were oriented radially, in the continuations of the direction of the straight line connecting the nest to the foraging holes (S2, S3, S4 and S5 Figs). Sixty percent of the new foraging trails observed during a visit departed directly from foraging holes, 35% originated from branching of existing trails and 5% were continuation of existing trails.

**Table 2:** Excavation Effort, Trail Construction Effort, and Area Covered by the Foraging Trails for the Four Nests Studied.

	Number of foraging holes			Number of physical trails			Cumulated length of physical trails (m)			Area covered (m^2^)		
Nest	WS^a^	DS^b^	Total	WS^a^	DS^b^	Total	WS^a^	DS^b^	Total	WS^a^	DS^b^	Total
N1	60	66	126	88	76	164	275.90	250.21	526.11	181.00	152.25	318.25
N2	53	51	104	87	121	208	223.90	529.80	753.70	142.75	278.75	406.50
N3	48	56	104	116	93	209	322.44	293.01	615.45	170.00	150.75	295.00
N4	83	42	125	170	82	252	458.48	275.23	733.71	257.00	155.50	366.25
Total	244	215	459	461	372	833	1280.72	1348.25	2628.97	1074.50	637.25	1346.00

^a^ Wet Season

^b^ Dry Season.

Foraging holes were located on average at 21.53 ± 10.89m (± SD, range: 3.19–63.74m) distance from the nests and the average length of the physical trails departing from these foraging holes was 4.72 ± 3.18m (± SD, range: 0.46–24.29m) (Table 3). Overall, there were differences among nests in the distance of foraging holes, both in the humid and in the dry season, as well as over the whole 8-month survey (Table 3). Nest 4 had always the most distant foraging holes. The length of the physical trails also differed among nests over the 8-month survey (Table 3). These differences were much more pronounced during the dry season than during the humid season.

**Table 3:** Mean ± SD of the Distance to the Nests of the Foraging Holes and Length of the Physical Trails.

	Distance of foraging holes to the nest (m)			
Nest	Wet Season	Dry Season	KS^✝^	All seasons
N1	18.55 ± 6.91	26.07 ± 10.25	<0.001	22.49 ± 9.56
N2	15.75 ± 5.99	19.82 ± 8.05	0.048	17.74 ± 7.33
N3	15.40 ± 5.84	15.90 ± 6.45	NS	15.67 ± 6.27
N4	29.07 ± 13.86	27.65 ±12.16	NS	28.59 ± 13.28
All nests	20.90 ± 11.25	22.24 ± 10.44	0.013	21.53 ± 10.89
	Length of the physical trails (m)			
Nest	Wet Season	Dry Season	KS^✝^	All seasons
N1	4.42 ± 2.56	3.78 ± 1.95	NS	4.12 ± 2.30
N2	4.07 ± 2.86	6.73 ± 4.64	<0.001	5.58 ± 4.18
N3	4.80 ± 3.38	4.80 ± 3.17	NS	4.80 ± 3.28
N4	4.59 ± 2.61	3.93 ± 2.17	0.010	4.37 ± 2.49
All nests	4.51 ± 2.84	4.98 ± 3.54	NS	4.72 ± 3.18

^✝^ Differences between the wet and humid season for each nest were tested with a Kolmogorov-Smirnov test (KS).

### Foraging activity

**Fig 2:**
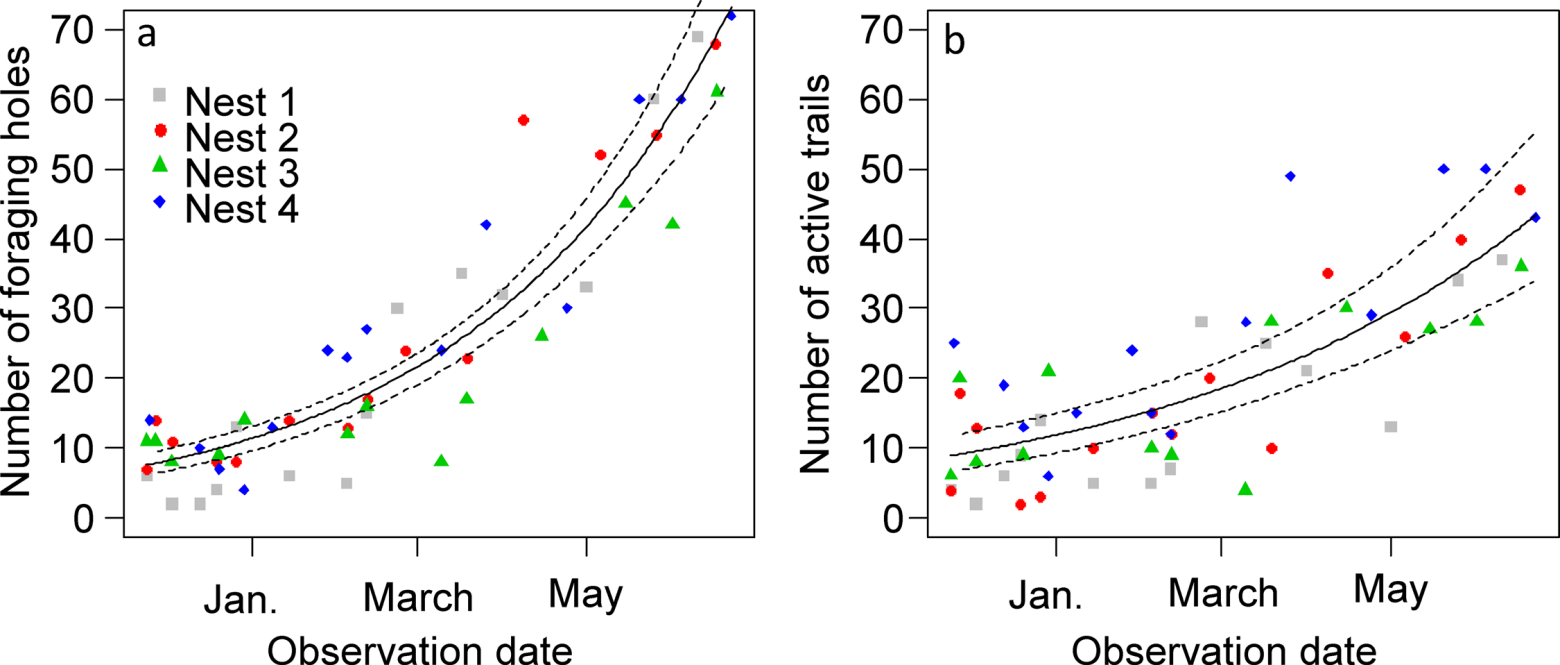
Assessment of foraging activity over the 8-month survey. (a) number of active foraging holes and (b) number of active physical trails observed at each visit. For each graph the bold continuous line shows the predictions of a quasipoisson Generalized Additive Mixed Model with nest entered as random variable; the dashed lines show the predictions ± CI_0.95_.

Both the number of active foraging holes (Fig 2A) and active foraging trails (Fig 2B) increased roughly exponentially throughout the 8-month survey. The number of active holes increased more rapidly than that of active trails, showing that, at least at the time of the visits, some of the holes could have foraging activity around them without necessarily having active foraging trails departing from them.

### Excavation and trail construction activity

**Fig 3:**
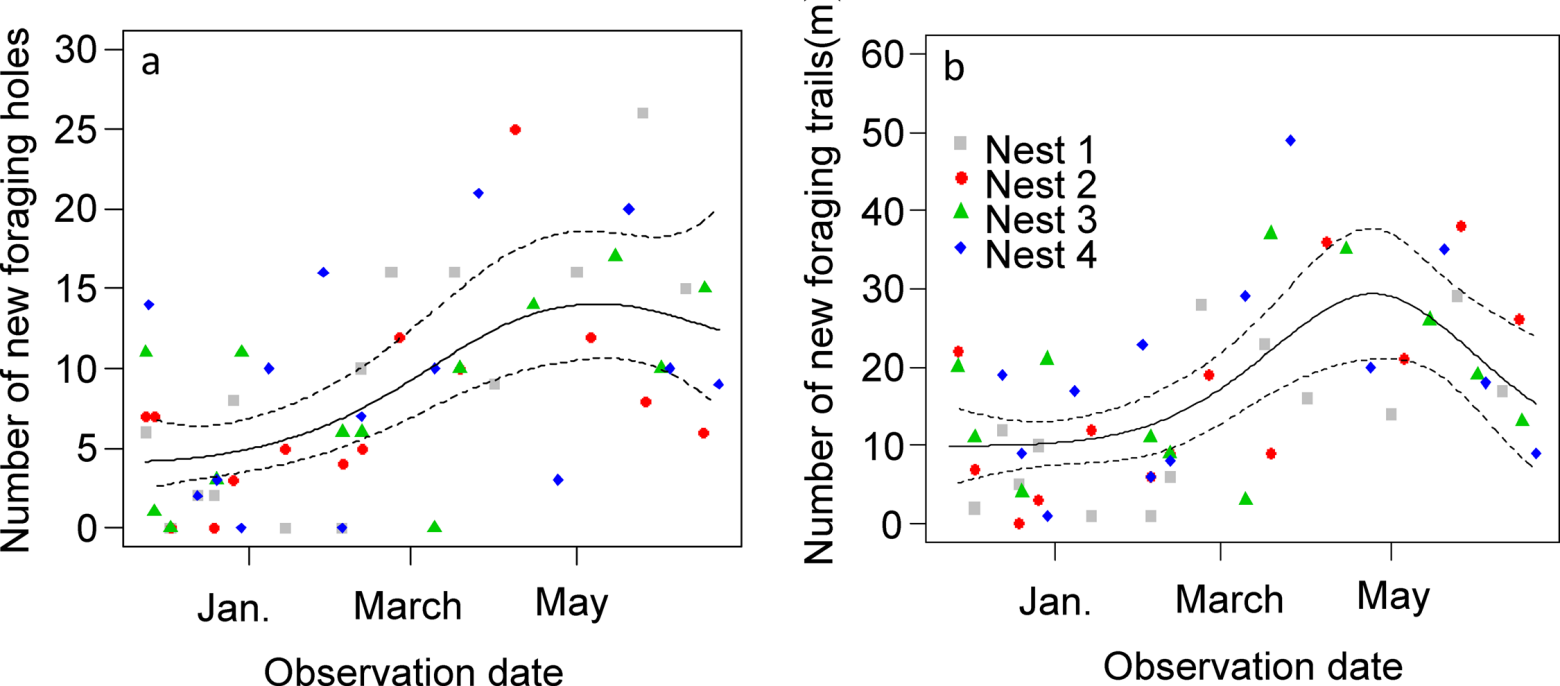
Assessment of the excavation and trail construction effort over the 8-month survey. (a) Excavation effort: number of new foraging holes counted at each visit; (b) Physical trail construction effort: number of new physical trails observed at each visit. For each graph the bold continuous line shows the predictions of a quasipoisson Generalized Additive Mixed Model with nest as random variable; the dashed lines show the predictions ± CI_0.95_.

Although the total number of new foraging holes appearing during the wet and dry season was comparable (Table 2) the rate at which they appeared differed between seasons (Fig 3A). Excavation activity increased from the beginning of the survey in November up to May and then was more or less stable until the end of the survey. The distance of the foraging holes to the nest increased significantly between the wet and the dry season for nest 1 and 2 whereas it was about the same for nest 3 and 4 (Table 3).

The pattern of change between the wet and the dry season in the number and cumulated length of new physical trails that appeared at each visit differed among the four nests surveyed. Whereas both the number (Table 2) and cumulated length of new physical trails (Table 2) increased for nest 2 between the wet and dry season, it decreased for all other nests (Table 2). The length of the foraging trails that were built increased significantly for nest 2 and decreased significantly for nest 4; it remained about the same for nest 1 and nest 3 (Table 3). The pattern of variation over time of the effort put in building physical trails was close to that observed for the excavation effort: it was stable until February, increased up to beginning of May and decreased thereafter (Fig 3B, S6 Fig).

### Use of the foraging network

**Fig 4:**
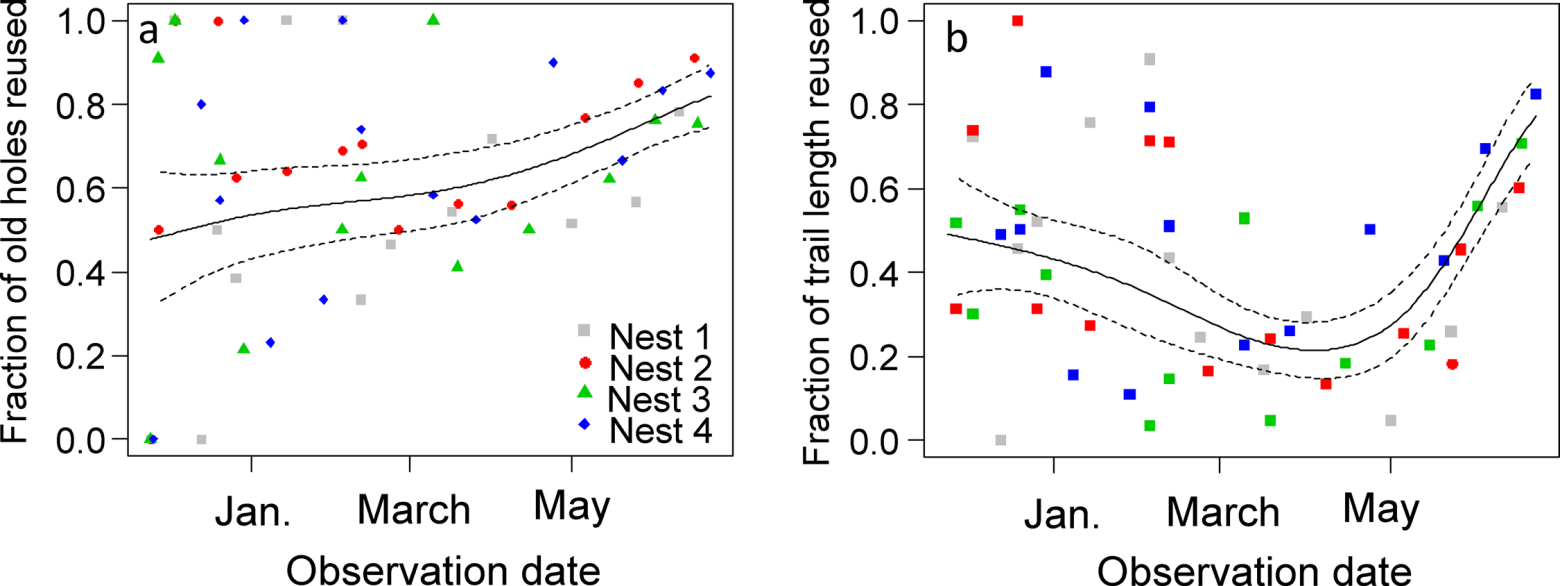
Dynamics of foraging network use over the 8-month survey. (a) Fraction of foraging holes and (b) fraction of total length of active physical trails reused at each visit. For each graph the bold continuous line shows the predictions of a quasibinomial Generalized Additive Mixed Model with nest as random variable; the dashed lines show the predictions ± CI_0.95._

The fraction of old foraging holes reused was highly variable during the wet season. Nevertheless, it increased at a slightly higher rate from the beginning of the dry season in April until the end of the survey (Fig 4A). When a foraging hole was active at a visit it was active at the next visit in 81% (N = 647) of the cases; in 9% of the cases it became inactive, and in 10% of the cases it was closed by ants. A closed hole remained closed at the next visit in 89% (N = 363) of the cases; in 9% of the cases it reopened and became active again. In the same way as for foraging holes, the fraction of old trail length reused was highly variable at the beginning of the survey. Nonetheless, it is clear from Fig 4B that it decreased slowly until the beginning of the dry season in April and then increased abruptly (Fig 4B).

### Distribution of foraging effort around the nest

The foraging activity of nest 1 was strongly concentrated in the W-SW sector of the nest whereas that of nest 4 was concentrated in the NE sector (Fig 1). The foraging activity of nest 2 and nest 3 on the other hand was evenly spread around the nest. The Hotelling confidence ellipse of nest 2 and 3 shows that foraging activity was slightly biased in the SE direction for both nests. Overall, there was no indication of a pattern of clockwise or counter-clockwise rotation of foraging activity around the nests over time (S7 Fig).

## Discussion

Overall our study shows that, as an ant collecting exclusively monocotyledons, *A*. *bisphaerica* build more ephemeral trails to collect their resources than the *Atta* species exploiting dicotyledons. Despite the seemingly less patchy distribution of the resources collected by grass cutting ants compared to leaf-cutting ant species, the foraging network of *A*. *bisphaerica* proved to be highly dynamic and few sections of the foraging trails were used for long periods of time. *A*. *bisphaerica* foraging workers rapidly shifted the location of their foraging activity, probably in order to choose the most profitable patches of grass. Our study also shows that the organization of the foraging networks can be strongly influenced by the particular features of the landscape surrounding the nests, in particular by the wet parts of the pastures in which the nests were located.

The foraging trails of the studied nests of *A*. *bisphaerica* were relatively short compared to other leaf-cutting ant species (e.g. *A*. *cephalotes* in which they can reach up to 100 meters [25]). As in *A*. *sexdens* [25] and *A*. *laevigata* [26] most of the foraging network consisted of underground tunnels. Although the initial cost of tunnel excavation should be higher than that of the construction of physical trails, the cost of their maintenance should be smaller than that of physical trails [25], particularly in the humid season because of the continuous growth of the vegetation that has to be removed from the physical trails. In addition, underground tunnels can offer some protection to grass-cutting ants which can be exposed to high temperatures and solar radiation intensities in pastures compared to leaf-cutting ants inhabiting tropical forests [41]. Compared to leaf-cutting ants [8] however, the cost of construction and maintenance of physical trails in grass-cutting ants should be offset by the fact that the grass cut by ants is probably transported inside the nest and used as substrate for their symbiotic fungus. In leaf-cutting ant species inhabiting tropical forests on the other hand, trail construction and maintenance mainly involves the removal of tiny twigs and dry leaf litter, which are a poor food resource for the fungus. The cost of physical trail maintenance in *A*. *bisphaerica* may be further reduced by the possible inhibitory action of the trail pheromone on the growth of vegetation [42].

The development and reorganization of the foraging network of the four nests of *A*. *bisphaerica* studied mainly occurred through the excavation of new foraging holes and the construction of new trails departing from existing or newly opened foraging holes. Contrary to most leaf-cutting ant species in which trunk-trails can be used for months or even years [11], the use of the foraging networks in *A*. *bisphaerica* colonies was highly dynamic. Hence, during the humid season on average more than 40% of the foraging holes and 50% of the physical trails that were active at each survey were new. Network development through the branching or the lengthening of established physical trails was much less common than in other leaf-cutting ant species in which foraging networks are clearly dendritic [11, 25, 43]. The pattern of network development observed in *A*. *bisphaerica* suggests that the search for new resources in this species occurs mostly in the vicinity of new foraging holes, whereas in leaf-cutting ants it occurs mostly in the vicinity of established foraging trails [18, 40]. Whether the excavation of new underground tunnels and new foraging holes obey particular rules is an intriguing question that remains to be investigated.

In leaf-cutting ants the distribution of palatable host plants is the main factor shaping the trail system, especially when they exploit pioneer plant species [29]. Although pastures can appear at first sight fairly homogenous, they can be composed of patches of several grass species which are differently preferred by ants [44] and have different effects on the growth of their symbiotic fungus [45]. Given the high level of activity and the big size of the colonies studied, the patches of preferred grass exploited by ants should be rapidly depleted. Also, when given the choice, foraging workers probably prefer to cut young and fresh blades of grass that are less tough and contain more sap [46]. Once the best quality forage has been collected, ants probably shift to another location. Shifting could be further enhanced by the fact that grass can produce secondary compounds as a defense system deterring further attack by ants [47, 48]. The quality of the pasture, its density, growth and composition, can also depend on local variation in terrain morphology, e.g. the slope that determines the exposure, and on soil chemical and physical properties, in particular its moisture (as illustrated here for Nest 1) and its compaction due to trampling by cattle. If the grass collected by ants were distributed homogenously a consistent shift in the angular direction of the foraging effort of the colonies would have been expected [49], which was the case for none of the nests studied.

The foraging networks of Nest 1 and Nest 4 were not centered on their nest position but significantly biased in one direction. This happened because physical obstacles were present around the nests. The foraging effort of Nest 1 was directed in the southwest direction because of the continuous flow of water trickling from a water tank located in this direction, which allowed grass to grow even in the dry season, and that of Nest 4 extended mostly northeastward because of a flooded area on its west side. On the other hand, the distribution of the foraging effort of nests 2 and 3 was not significantly biased towards any direction, as indicated by the fact that the nest positions were included in the Hotelling confidence ellipses. Compared to other nests, the foraging holes of these two nests were also located at relatively short distances from their respective nests. This was probably due to their proximity, which prevented the expansion of their territory on one side. Territorial expansion was thus mainly achieved by building longer foraging trails in the direction opposed to the closest nest.

**Fig 5:**
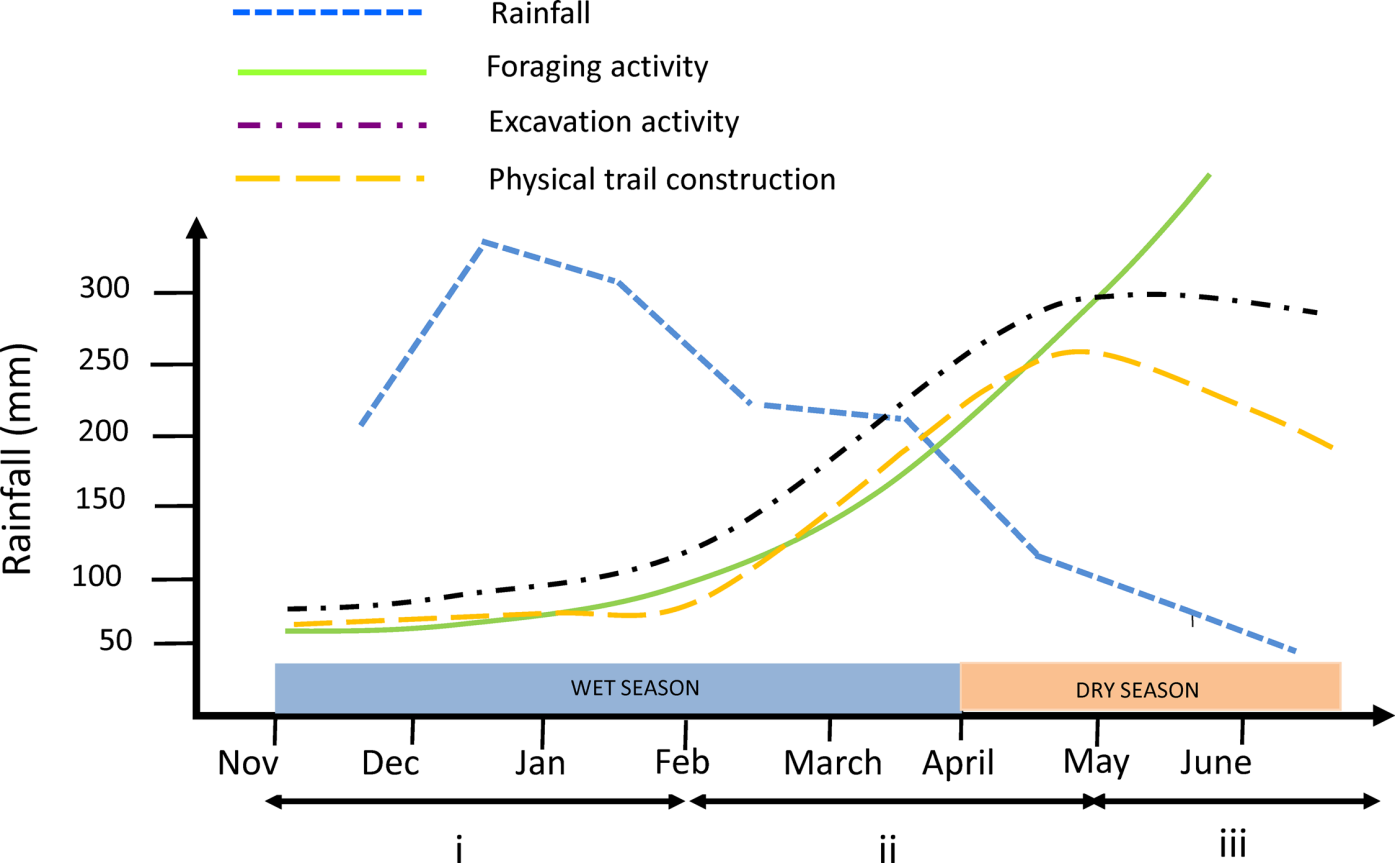
Diagram showing how foraging, excavation, and physical trail construction could be related to rainfall. Rainfall data are from the Brazilian Institute of Meteorology meteorological station of the city of Juiz de Fora (about 12 Km from the study site).

The change in weather conditions observed throughout the 8-month observation period is likely to have had a great influence on the construction, maintenance, and use of the foraging networks. Three different phases, which could be linked to the rainfall regime, can be distinguished in the activity of the colonies (Fig 5). The first phase corresponds to the first half of the rainy season (November 2009 to February 2010) and is characterized by low foraging activity, as well as low excavation and physical trail construction effort. Beginning in February, corresponding to the start of the second phase of foraging activity, the colonies increased the collection of plant material at a much higher rate and began to expand their foraging area. This coincides with the increase in fresh grass supply promoted by rain during the peak of the humid season (end of December–January). Excavation and trail construction effort began to increase in February, corresponding to the beginning of the second phase of foraging activity, and they kept increasing until May, resulting in an expansion of the foraging networks of the colonies. During this phase ants opened a lot of new foraging holes and built a lot of new foraging trails. Finally, during the third phase (May to June 2010), which corresponds to the beginning of the dry season, excavation effort leveled off and trail construction activity decreased. Foraging activity kept increasing however and one could hypothesize that it could do so because ants were using the infrastructures (tunnels and trails) built during the humid season. The underground tunnels built during the humid season should be preserved and, in any case, they should require few maintenance work. As for the physical trails, they should be maintained for longer period of time during the dry season because of the slow growth of the vegetation. When nest 1 was excluded there was a parallel increase over the survey period between the growth of the other colonies, assessed by the increase in the area of loose soil over the nests (Table 1), and the growth of foraging activity, assessed by the cumulated length of active physical trails (Table 2). This shows that the increase in the area of loose soil over the nests is a good measure of nest growth [50]. This may not be true for nest 1 however because ants of this nest had access to a permanent supply of fresh grass on their foraging area.

We hypothesize that the temporal variation of the structure and organization of the foraging networks of *A*. *bisphaerica* could be understood as the product of the interaction between two main factors: rainfall regime and colony growth. On the one hand, local rainfall variation (which is mainly linked to the alternation between the wet and dry season) is likely to have a direct influence on both soil resistance, and thus on tunnel excavation effort [51], and pasture development, and thus on the construction, maintenance and use of physical foraging trails. On the other hand, the growth of the colonies should imply a higher foraging effort, which could be achieved through the expansion of the foraging networks in the humid season and through the use of the network infrastructures built during the humid season in the dry season. At present however this scenario remains speculative as it is based on a survey covering a whole humid season and only part of a dry season of a single year; the validity of this scenario should be confirmed by a survey extending over several successive years.

In *A bisphaerica*, compared to leaf-cutting ants, network expansion seems to occur mostly under the soil surface, as suggested by the fact that the distance of newly excavated foraging holes from the nests kept increasing over time. The underground foraging network formed by these tunnels is probably as complex as the superficial networks of trunk-trails described in leaf-cutting ants and would deserve to be investigated.

## Supporting Information

S1 Fig General view of the field site. (TIF)

S2 Fig Map of the foraging trails of Nest 1 on the last day of visit. Each color corresponds to the survey date at which the foraging holes and trails were first observed.
(TIF)

S3 Fig Map of the foraging trails of Nest 2 on the last day of visit. Each color corresponds to the survey date at which the foraging holes and trails were first observed.
(TIF)

S4 Fig Map of the foraging trails of Nest 3 on the last day of visit. Each color corresponds to the survey date at which the foraging holes and trails were first observed.
(TIF)

S5 Fig Map of the foraging trails of Nest 4 on the last day of visit. Each color corresponds to the survey date at which the foraging holes and trails were first observed.
(TIF)

S6 Fig Assessment of the effort put in physical trail construction over the 8-month survey. Cumulated length of new physical foraging trails. The bold continuous line shows the predictions of a Generalized Additive Mixed Model with nest as random variable; the dashed lines show the predictions ± CI_0.95_.
(TIFF)

S7 Fig Direction of the mean vectors of the end point of foraging trails at each visit. The angles are measured anticlockwise, with 0° corresponding to the East. Big and small stars indicate the visits for which the direction of the end points of the foraging trails were significantly concentrated (at *P*<0.01 and 0.05>*P*>0.01, respectively) around the direction of the mean vector (Rayleigh test). If there was a consistent shift in one direction in the foraging activity of a colony the value of the direction of the mean vectors should increase or decrease monotonically.
(TIF)
