# Supplementary figures and images for: Spatio-Temporal Dynamics of Foraging Networks in the Grass-Cutting Ant Atta bisphaerica Forel, 1908 (Formicidae, Attini)
Source: PLoS One. 2016 Jan 11;11(1):e0146613. doi: 10.1371/journal.pone.0146613 (PMC4720121; doi:10.1371/journal.pone.0146613)

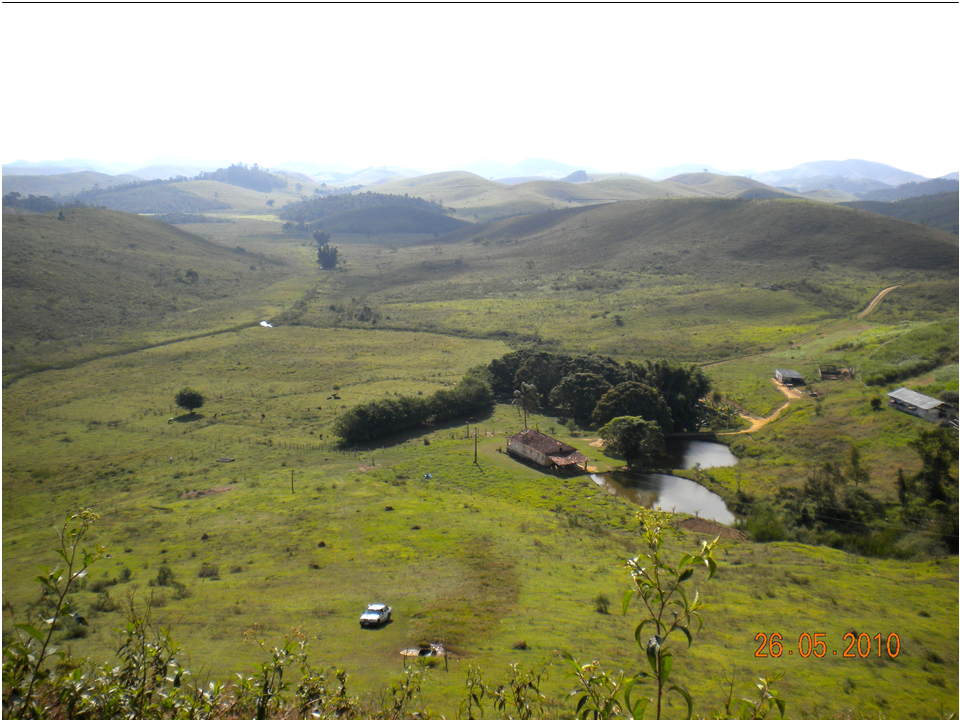

Supplement: S1 Fig — (TIF) [file pone.0146613.s001.tif]

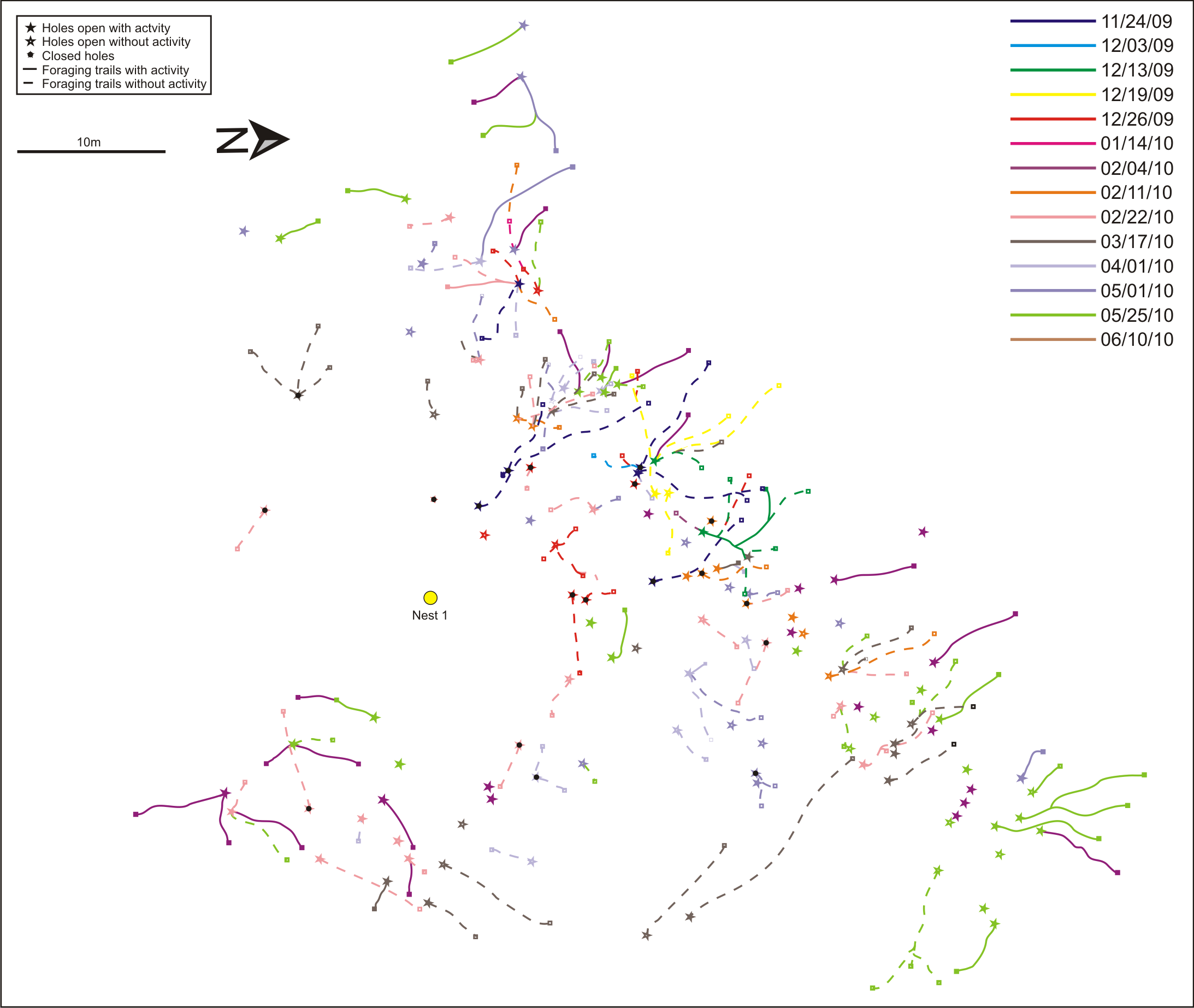

Supplement: S2 Fig — Each color corresponds to the survey date at which the foraging holes and trails were first observed. (TIF) [file pone.0146613.s002.tif]

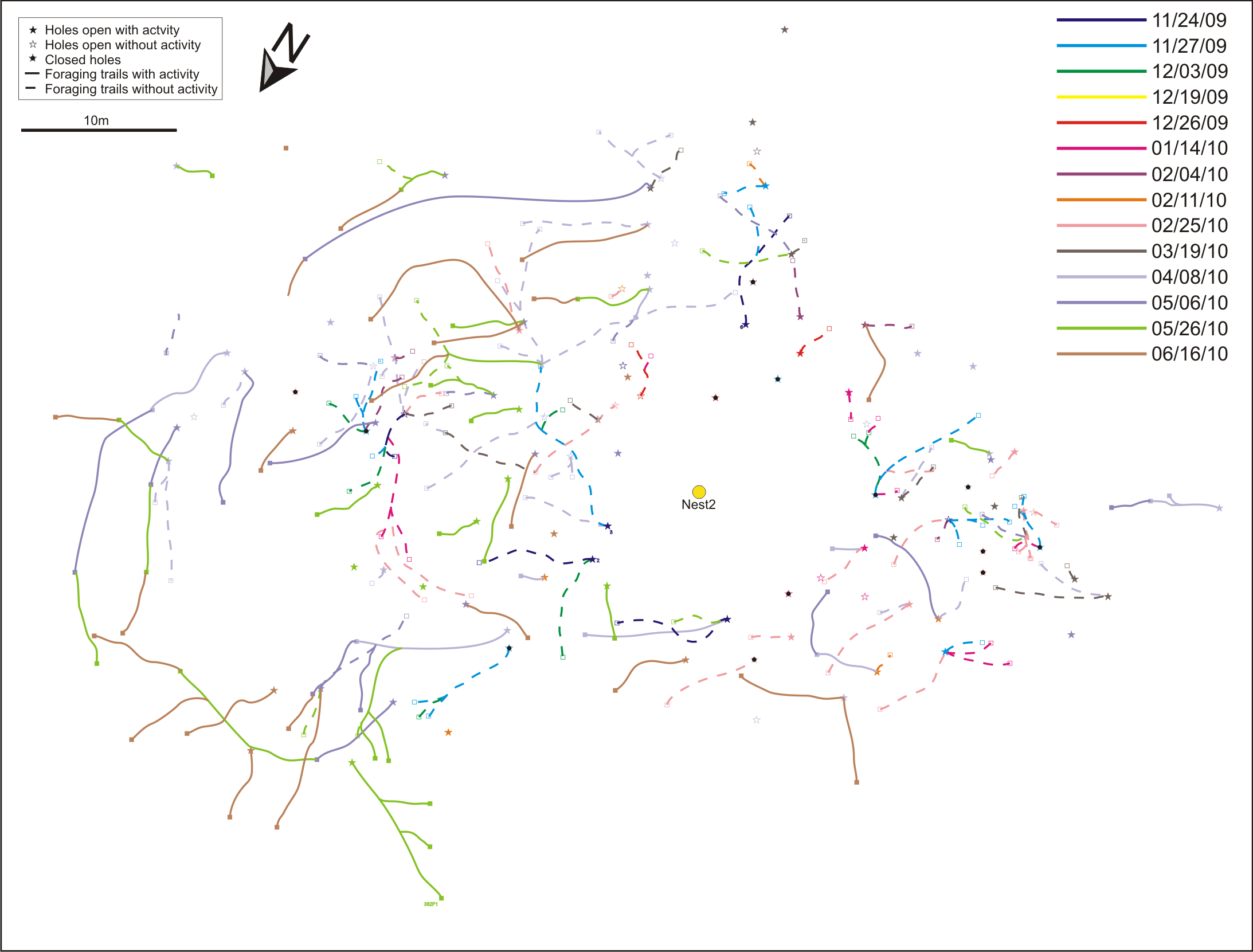

Supplement: S3 Fig — Each color corresponds to the survey date at which the foraging holes and trails were first observed. (TIF) [file pone.0146613.s003.tif]

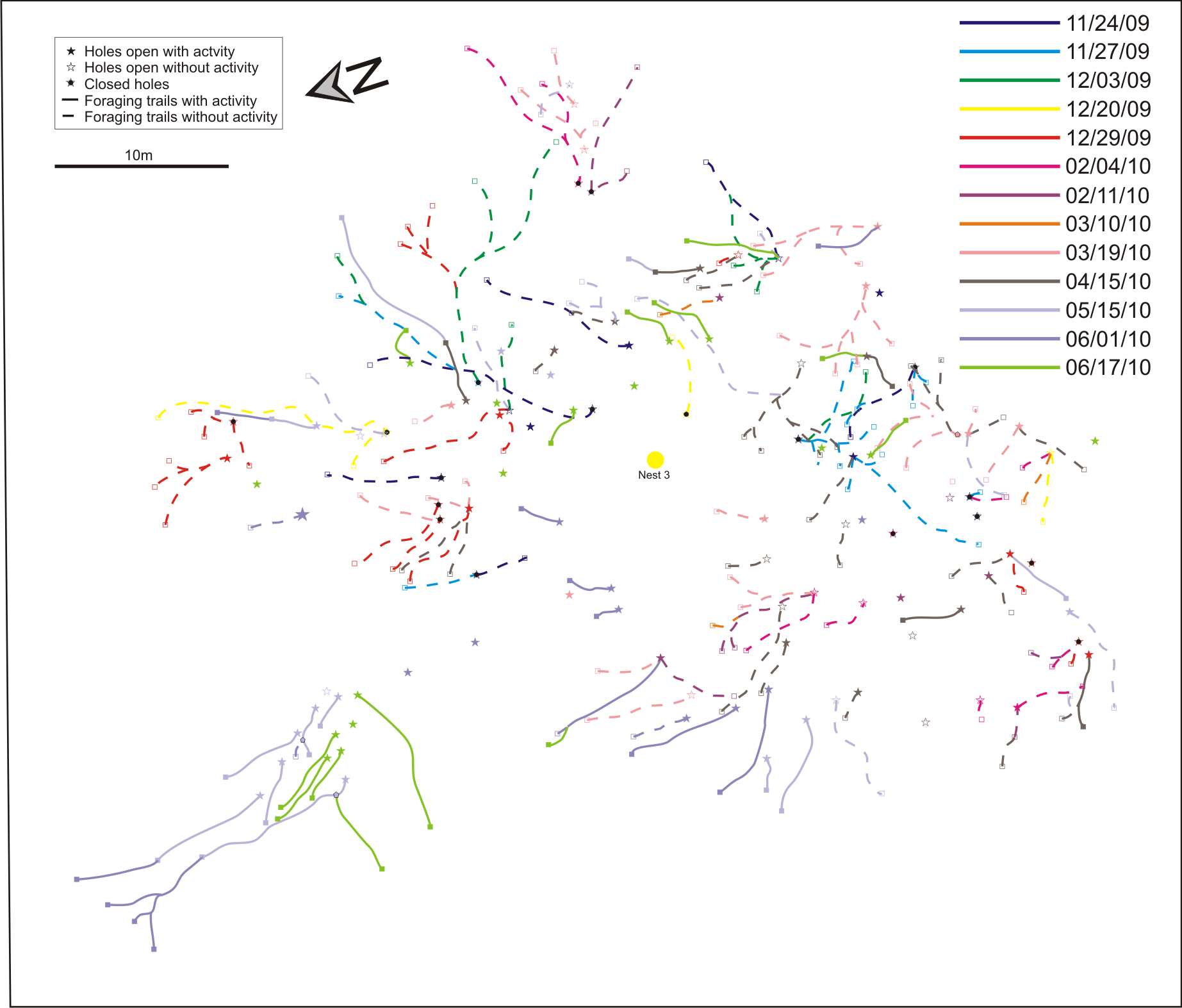

Supplement: S4 Fig — Each color corresponds to the survey date at which the foraging holes and trails were first observed. (TIF) [file pone.0146613.s004.tif]

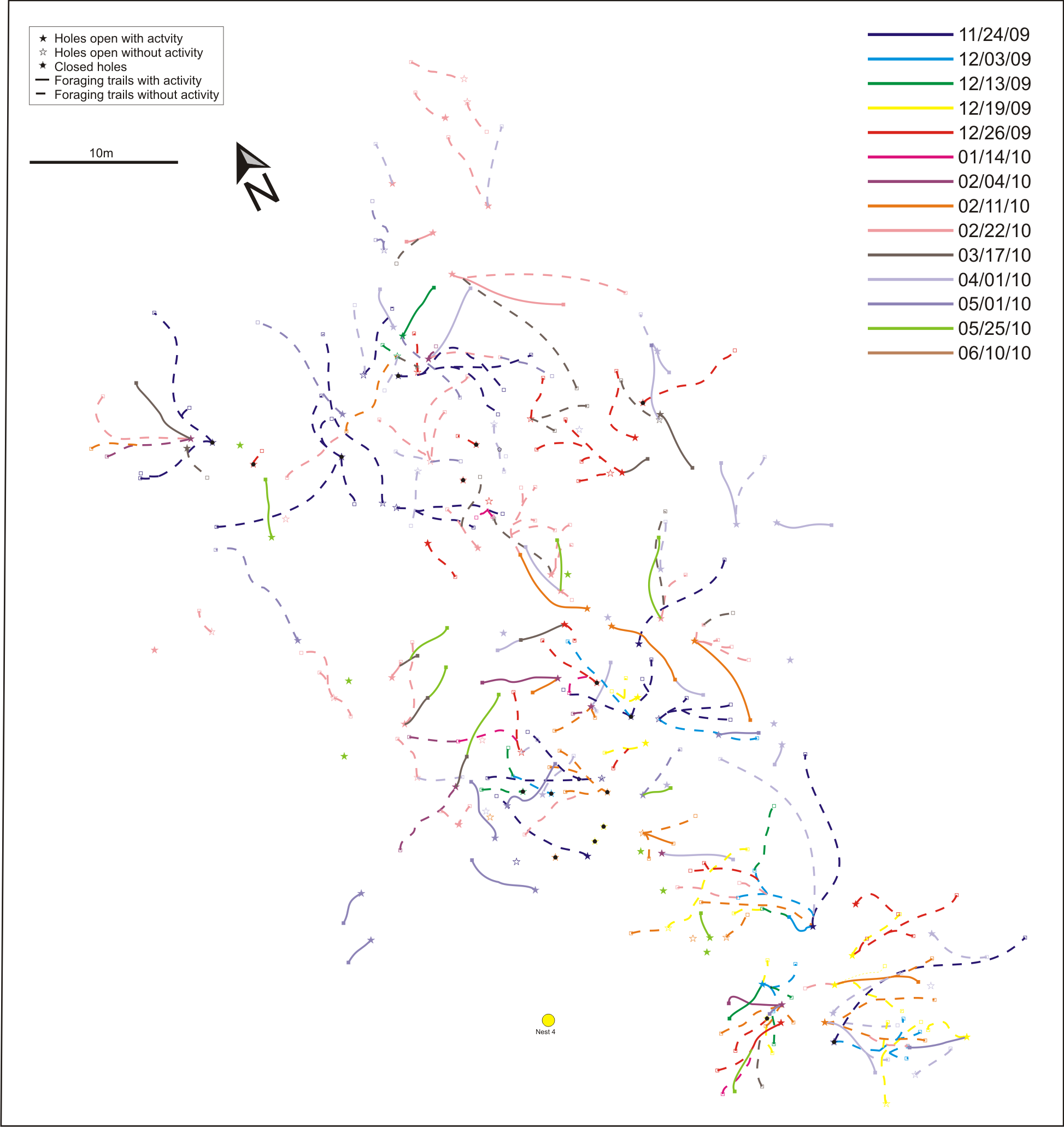

Supplement: S5 Fig — Each color corresponds to the survey date at which the foraging holes and trails were first observed. (TIF) [file pone.0146613.s005.tif]

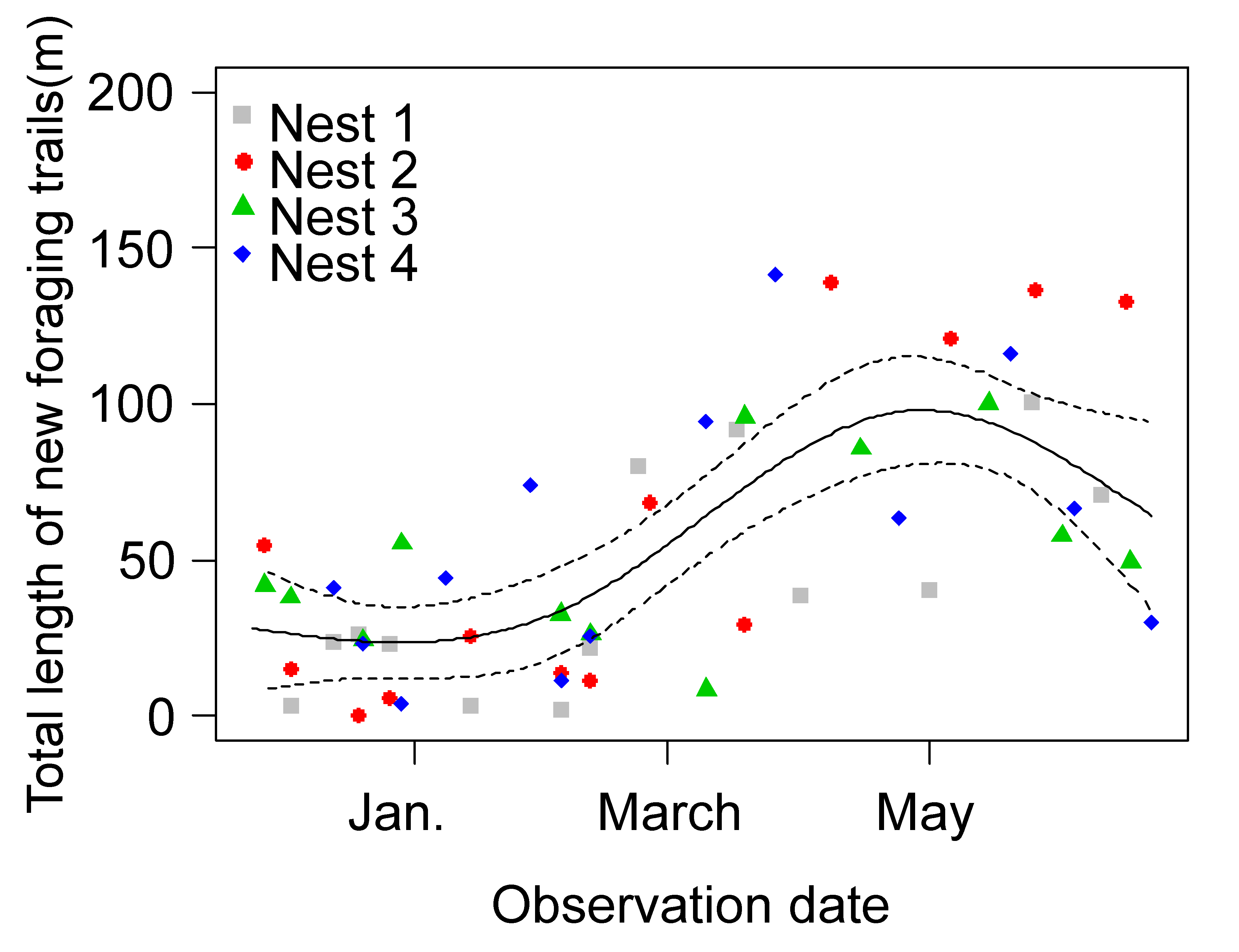

Supplement: S6 Fig — Cumulated length of new physical foraging trails. The bold continuous line shows the predictions of a Generalized Additive Mixed Model with nest as random variable; the dashed lines show the predictions ± CI0.95. (TIFF) [file pone.0146613.s006.tiff]

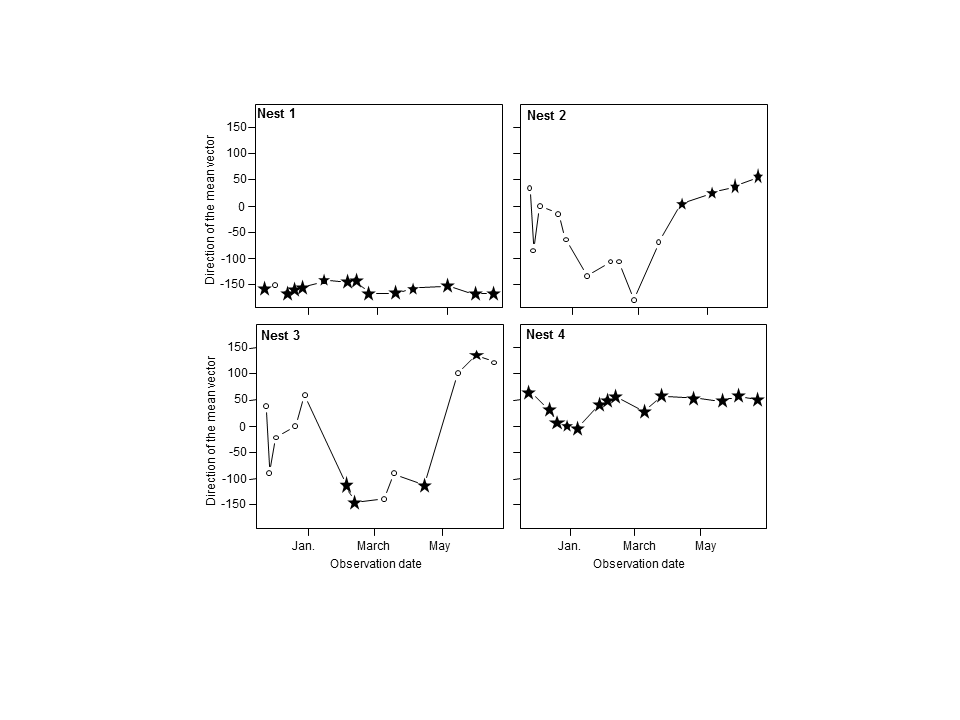

Supplement: S7 Fig — The angles are measured anticlockwise, with 0° corresponding to the East. Big and small stars indicate the visits for which the direction of the end points of the foraging trails were significantly concentrated (at P<0.01 and 0.05>P>0.01, respectively) around the direction of the mean vector (Rayleigh test). If there was a consistent shift in one direction in the foraging activity of a colony the value of the direction of the mean vectors should increase or decrease monotonically. (TIF) [file pone.0146613.s007.tif]
